# Comparison of clip and needle electrodes for multifrequency bioimpedance analysis in anesthetized dogs

**DOI:** 10.1371/journal.pone.0355338

**Published:** 2026-08-06

**Authors:** Jun Tamura, Takaharu Itami, Keiko Kato, Chihiro Sugita, Norihiko Oyama, Kazuto Yamashita

**Affiliations:** 1 Faculty of Veterinary Medicine, Veterinary Teaching Hospital, Hokkaido University, Sapporo, Hokkaido, Japan; 2 Department of Veterinary Science, School of Veterinary Medicine, Rakuno Gakuen University, Ebetsu, Hokkaido, Japan; Yarmouk University, JORDAN

## Abstract

Multifrequency bioimpedance analysis provides resistance, reactance, and phase angle measurements that are used to characterize body fluid distribution and cellular electrical properties. However, studies reporting on body composition assessment using multifrequency bioimpedance analysis in dogs remain limited. Therefore, we aimed to evaluate the measurement characteristics of trunk multifrequency bioimpedance analysis in dogs and assess the influence of body position and electrode type and placement. We anesthetized seven adult Beagle dogs and performed trunk multifrequency bioimpedance analysis at 5, 50, and 250 kHz using clip and needle electrodes in sternal and left lateral recumbency at three electrode paths. For each condition, 150 repeated measurements were obtained and summarized as a mean value. Repeatability and inter-individual variability were assessed using coefficients of variation. Agreement between electrode types was assessed using Bland–Altman analysis and correlation analysis, and the effects of electrode type and body position were evaluated using linear mixed-effects models with dogs as a random effect. Measurements were feasible under all conditions and showed low intra-individual variability. Inter-individual variability was generally 10–15%, but higher variability was observed for reactance and phase angle at low frequencies. Mean biases between clip and needle electrodes were small for most parameters; however, reactance and phase angle at 5 kHz showed weaker correlations and wider limits of agreement. Across models, between-dog differences accounted for most variability, whereas body position influenced several parameters when the stifle was included in the electrode path. These data indicate that clip electrodes yield measurements with small mean biases relative to those obtained using needle electrodes under controlled conditions in anesthetized dogs; however, the limits of agreement varied among parameters, and wider differences observed at low frequencies suggest that the two electrode types should not be considered fully interchangeable. Accordingly, electrode placement and body position should be standardized for experimental comparison and carefully controlled when monitoring longitudinal changes within individual dogs.

## Introduction

Bioimpedance analysis is a minimally invasive technique that evaluates the electrical impedance of biological tissues and provides parameters such as resistance, reactance, and phase angle [1,2]. Resistance reflects opposition to electrical current flow through conductive body fluids and is influenced by water amount and distribution of water. Reactance and phase angle reflect capacitive properties related to cell membranes and are influenced by cellular integrity and tissue composition.

Compared with single-frequency bioimpedance analysis, multifrequency bioimpedance analysis (MF-BIA), which measures impedance across a range of frequencies, provides more comprehensive information regarding body fluid distribution and cellular function [3–5]. At low frequencies, electrical current primarily travels through the extracellular space because cell membranes act as insulators. Therefore, resistance at low frequencies mainly reflects extracellular fluid status. At higher frequencies, the current penetrates cell membranes, enabling the assessment of both extracellular and intracellular compartments.

In human medicine, MF-BIA using clip electrodes is widely applied as a non-invasive and cost-effective tool for assessing body composition, fluid balance, nutritional status, and prognosis in various clinical settings, including chronic kidney disease [6,7], heart failure [7], sepsis [8], cancer [9], and malnutrition [5]. In domestic dogs (*Canis lupus familiaris*), single-frequency bioimpedance analysis using various types of electrodes, including needles, clips, adhesives, and contact electrodes, is commonly used to estimate total body water, body fat composition, and fluid redistribution after blood donation [10–16]. However, studies reporting on body composition assessment using MF-BIA in dogs remain limited [17,18]. Clinically evident peripheral oedema has been reported in critically ill dogs with systemic inflammatory conditions, highlighting the potential clinical importance of objectively assessing alterations in body fluid distribution [19]. Nevertheless, fundamental data on the characteristics of MF-BIA measurements in dogs and the influence of measurement conditions, such as electrode placement site, electrode type, and body position are lacking.

The objectives of this study were (i) to characterize trunk MF-BIA measurements in anesthetized dogs and quantify repeatability and inter-individual variability across various body positions and electrode placement sites, and (ii) to evaluate clip electrodes as a non-invasive attachment method in comparison with needle electrodes, (which are commonly used in experimental settings), to support future clinical applications. We hypothesized that clip electrodes would demonstrate acceptable repeatability and generally good agreement with needle electrodes under controlled anesthetized conditions and that electrode placement and body position would contribute to between-condition differences.

## Materials and methods

### Ethics statement

This study was approved by the Animal Care and Use Committee of Rakuno Gakuen University (approval No.: VH23B9) and was reported in accordance with the ARRIVE guidelines 2.0.

### Animals

Seven intact adult beagle dogs (4 males and 3 females), aged 7–9 years and weighing 8.9–15.5 kg [12.9 ± 2.2 kg (mean ± standard deviation)], were included. The dogs were obtained from a licensed vendor (Hokudo Co., Ltd., Japan) and were considered clinically healthy based on physical examination, complete blood cell count, and biochemistry panel, except for mild hypoalbuminemia in two dogs and elevated alanine aminotransferase in one dog. Food was withheld for at least 12 h before drug administration; however, the dogs had free access to water 30 min before treatment.

### Anesthesia and monitoring

Anesthesia was induced by mask induction using sevoflurane in oxygen, followed by endotracheal intubation. Anesthesia was maintained with sevoflurane in oxygen (2.5%). The dogs were positioned in sternal recumbency on a conductive mat and left undisturbed for 30 min to allow stabilization and static electricity discharge.

Rocuronium was administered (0.5 mg/kg IV) followed by constant rate infusion (0.5 mg/kg/h) via an intravenous catheter placed in the left cephalic vein. Physiologic variables were monitored using a multiparameter anesthetic monitor. Mechanical ventilation was adjusted to maintain end-tidal carbon dioxide at 35–40 mmHg (4.7–5.3 kPa). Lactated Ringer’s solution (3 mL/kg/h) was administered intravenously throughout anesthesia. After completion of measurements, sugammadex (4 mg/kg IV) was administered, and the dogs recovered uneventfully.

### MF-BIA measurement protocol

MF-BIA was performed using a tetrapolar bioimpedance spectrometer (InBody M20, InBody Japan Inc.), which measured resistance, reactance, and phase angle at 5, 50, and 250 kHz. Measurements were conducted in two body positions (sternal and left lateral recumbency) on a non-conductive silicone mat using clip electrodes and needle electrodes.

To minimize limb contact in lateral recumbency, a cotton towel was placed between the left and right limbs to avoid contact and crossing. Measurements followed a fixed sequence: sternal recumbency with clip electrodes, lateral recumbency with clip electrodes, sternal recumbency with needle electrodes, and lateral recumbency with needle electrodes. A 15-min stabilization period was allowed after each change in body position. Hair at the electrode sites was clipped closely and cleaned with alcohol. For clip electrodes, conductive electrocardiography cream (Keratin Cream; Fukuda Denshi) was applied to improve contact. Needle electrodes consisted of 25-gauge needles inserted subcutaneously to a length of 1 cm.

For each condition, measurements were obtained sequentially across three electrode paths: from the right elbow to the right stifle (ESt), from the level of the first thoracic spinous process to the right stifle (TSt), and from the vertex of the head to the level of the sacral spinous process (HSc). For each path, MF-BIA was measured 150 times at 50 ms intervals and summarized as the mean value for analysis.

### Cole–Cole modeling and data quality control

Based on the Cole–Cole model, resistance at zero frequency (R0), resistance at infinite frequency (Rinf), maximum reactance (Xmax), and maximum phase angle (PAmax) were calculated [5]. Measurements were excluded if the resistance at the center of the Cole–Cole circle was not within the range defined by resistance values at 5 and 250 kHz [5]. Randomization and blinding were not applied because all animals underwent measurements in a fixed sequence under identical conditions, and outcomes were recorded directly from instrument output.

### Statistical analysis

Descriptive data are presented as mean ± standard deviation. Repeatability was assessed by calculating the coefficient of variation from each set of 150 repeated measurements (standard deviation/mean). Inter-individual variability was evaluated using the coefficient of variation calculated across dogs for each condition. Agreement between clip and needle electrodes was assessed using Bland–Altman analysis. For each dog, body position, and electrode path, the mean value obtained with clip electrodes was paired with the corresponding mean value obtained with needle electrodes. Differences were calculated as clip minus needle measurements. The bias, 95% limits of agreement, their corresponding 95% confidence intervals (CI), and percentage limits of agreement were calculated. Correlation coefficients and associated *P* values were estimated using regression analysis. Linear mixed-effects models were used to evaluate fixed effects (electrode type and body position) on MF-BIA variables, with dogs included as a random effect. A *P* value <0.05 was considered statistically significant. Analyses were performed using JMP Pro version 18.2.0 (SAS Institute Inc., Cary, NC, USA).

## Results

A total of 1800 measurements were completed for each dog (150 measurements × 3 electrode paths × 4 conditions). MF-BIA measurements were performed at 53.3 ± 10.5, 69.0 ± 10.7, 84.0 ± 11.5, and 103.0 ± 13.9 min after endotracheal intubation in the sternal recumbency with clip electrodes, lateral recumbency with clip electrodes, sternal recumbency with needle electrodes, and lateral recumbency with needle electrodes groups, respectively. Because the resistance at the center of the Cole–Cole circle fell outside the range defined by the resistance values at 5 and 250 kHz, the ESt and TSt data from one dog in the sternal recumbency with clip electrodes group and the TSt data from a different dog in the lateral recumbency with clip electrodes group were excluded from the analysis. MF-BIA parameters and analysis status for individual dogs are provided in S1 Table.

Repeatability and inter-individual variability are summarized in Tables 1 and 2.

**Table 1:** Repeatability of MF-BIA measurements expressed as coefficients of variation (%).

Electrode type	Body position	Electrode path	Resistance 5 kHz	Resistance 50 kHz	Resistance 250 kHz
Clip	Sternal	ESt	0.07	0.08	0.08
Clip	Sternal	TSt	0.10	0.11	0.16
Clip	Sternal	HSc	0.12	0.13	0.14
Clip	Lateral	ESt	0.14	0.13	0.12
Clip	Lateral	TSt	0.10	0.10	0.10
Clip	Lateral	HSc	0.11	0.11	0.12
Needle	Sternal	ESt	0.07	0.06	0.06
Needle	Sternal	TSt	0.09	0.09	0.10
Needle	Sternal	HSc	0.11	0.11	0.12
Needle	Lateral	ESt	0.13	0.13	0.12
Needle	Lateral	TSt	0.10	0.10	0.09
Needle	Lateral	HSc	0.10	0.10	0.11
**Electrode type**	**Body position**	**Electrode path**	**Reactance 5 kHz**	**Reactance 50 kHz**	**Reactance 250 kHz**
Clip	Sternal	ESt	0.25	0.13	0.08
Clip	Sternal	TSt	0.57	0.19	0.17
Clip	Sternal	HSc	0.64	0.19	0.19
Clip	Lateral	ESt	0.38	0.26	0.20
Clip	Lateral	TSt	0.32	0.22	0.20
Clip	Lateral	HSc	0.47	0.16	0.14
Needle	Sternal	ESt	0.25	0.14	0.10
Needle	Sternal	TSt	0.36	0.14	0.14
Needle	Sternal	HSc	0.39	0.17	0.16
Needle	Lateral	ESt	0.37	0.24	0.19
Needle	Lateral	TSt	0.32	0.20	0.19
Needle	Lateral	HSc	0.19	0.17	0.16
**Electrode type**	**Body position**	**Electrode path**	**Phase angle 5 kHz**	**Phase angle 50 kHz**	**Phase angle 250 kHz**
Clip	Sternal	ESt	0.36	0.11	0.00
Clip	Sternal	TSt	0.54	0.32	0.23
Clip	Sternal	HSc	0.44	0.05	0.13
Clip	Lateral	ESt	0.32	0.18	0.11
Clip	Lateral	TSt	0.23	0.10	0.24
Clip	Lateral	HSc	0.28	0.35	0.22
Needle	Sternal	ESt	0.26	0.28	0.03
Needle	Sternal	TSt	0.19	0.21	0.08
Needle	Sternal	HSc	0.22	0.05	0.13
Needle	Lateral	ESt	0.68	0.36	0.10
Needle	Lateral	TSt	0.44	0.28	0.16
Needle	Lateral	HSc	0.72	0.01	0.12

**Table 2:** Inter-individual variability of MF-BIA measurements expressed as coefficients of variation (%).

Electrode type	Body position	Electrode path	Resistance 5 kHz	Resistance 50 kHz	Resistance 250 kHz
Clip	Sternal	ESt	11.45	11.07	10.34
Clip	Sternal	TSt	11.87	12.58	12.21
Clip	Sternal	HSc	8.54	8.82	9.93
Clip	Lateral	ESt	11.62	11.84	11.22
Clip	Lateral	TSt	16.17	15.45	15.32
Clip	Lateral	HSc	12.07	14.24	16.64
Needle	Sternal	ESt	10.19	10.97	11.23
Needle	Sternal	TSt	14.64	14.95	14.83
Needle	Sternal	HSc	13.41	14.29	16.68
Needle	Lateral	ESt	12.66	13.02	12.77
Needle	Lateral	TSt	16.49	16.84	16.99
Needle	Lateral	HSc	10.28	11.14	13.26
**Electrode type**	**Body position**	**Electrode path**	**Reactance 5 kHz**	**Reactance 50 kHz**	**Reactance 250 kHz**
Clip	Sternal	ESt	24.08	16.91	14.80
Clip	Sternal	TSt	24.08	15.01	17.05
Clip	Sternal	HSc	22.35	12.88	11.62
Clip	Lateral	ESt	38.59	16.42	15.13
Clip	Lateral	TSt	38.77	21.11	19.43
Clip	Lateral	HSc	11.74	10.01	12.43
Needle	Sternal	ESt	10.65	10.08	11.72
Needle	Sternal	TSt	20.54	17.07	19.68
Needle	Sternal	HSc	17.88	13.60	13.38
Needle	Lateral	ESt	11.74	14.03	12.99
Needle	Lateral	TSt	17.85	17.82	19.09
Needle	Lateral	HSc	14.27	12.65	13.44
**Electrode type**	**Body position**	**Electrode path**	**Phase angle 5 kHz**	**Phase angle 50 kHz**	**Phase angle 250 kHz**
Clip	Sternal	ESt	17.30	9.81	7.43
Clip	Sternal	TSt	22.75	8.62	6.48
Clip	Sternal	HSc	17.92	11.53	9.01
Clip	Lateral	ESt	31.08	8.56	7.05
Clip	Lateral	TSt	28.80	11.69	9.51
Clip	Lateral	HSc	19.96	15.22	10.52
Needle	Sternal	ESt	11.03	4.91	1.79
Needle	Sternal	TSt	16.08	8.39	11.66
Needle	Sternal	HSc	14.98	13.72	12.94
Needle	Lateral	ESt	10.20	7.00	4.44
Needle	Lateral	TSt	12.36	8.03	8.54
Needle	Lateral	HSc	13.36	13.28	12.87

Intra-individual coefficients of variation were <1% for resistance, reactance, and phase angle under all conditions. Inter-individual coefficients of variation for ESt and HSc were generally around 10–15%, with higher values observed for reactance at 5 and 50 kHz and for phase angle at 5 kHz. For TSt, inter-individual coefficients of variation for resistance and reactance frequently exceeded 15%, particularly in lateral recumbency.

MF-BIA measurements across body position, electrode path, and electrode type are shown in Table 3.

**Table 3:** MF-BIA measurements obtained in anesthetized dogs.

Electrode type	Body position	Electrode path	Resistance 5 kHz (Ω)	Resistance 50 kHz (Ω)	Resistance 250 kHz (Ω)
Clip	Sternal	ESt	254.4 ± 29.1	217.5 ± 24.1	164.7 ± 17.0
Clip	Sternal	TSt	202.2 ± 24.0	173.9 ± 21.9	135.5 ± 16.5
Clip	Sternal	HSc	237.6 ± 20.3	207.4 ± 18.3	170.7 ± 17.0
Clip	Lateral	ESt	226.3 ± 26.3	194.5 ± 23.0	151.2 ± 17.0
Clip	Lateral	TSt	188.7 ± 30.5	160.3 ± 24.8	129.0 ± 19.8
Clip	Lateral	HSc	231.8 ± 28.0	203.7 ± 29.0	168.5 ± 28.0
Needle	Sternal	ESt	255.3 ± 26.0	218.8 ± 24.0	165.5 ± 18.6
Needle	Sternal	TSt	196.4 ± 28.7	169.5 ± 25.3	133.9 ± 19.9
Needle	Sternal	HSc	239.9 ± 32.2	211.1 ± 30.2	176.5 ± 29.4
Needle	Lateral	ESt	224.2 ± 28.4	192.9 ± 25.1	151.8 ± 19.4
Needle	Lateral	TSt	185.1 ± 30.5	159.4 ± 26.8	130.1 ± 22.1
Needle	Lateral	HSc	236.1 ± 24.3	207.4 ± 23.1	173.1 ± 23.0
**Electrode type**	**Body position**	**Electrode path**	**Reactance 5 kHz (Ω)**	**Reactance 50 kHz (Ω)**	**Reactance 250 kHz (Ω)**
Clip	Sternal	ESt	13.6 ± 3.3	40.0 ± 6.8	42.3 ± 6.3
Clip	Sternal	TSt	11.1 ± 2.7	29.9 ± 4.5	31.6 ± 5.4
Clip	Sternal	HSc	13.0 ± 2.9	30.1 ± 3.9	33.0 ± 3.8
Clip	Lateral	ESt	14.0 ± 5.4	33.9 ± 5.6	34.3 ± 5.2
Clip	Lateral	TSt	13.2 ± 5.1	26.4 ± 5.6	25.2 ± 4.9
Clip	Lateral	HSc	11.4 ± 1.3	28.4 ± 2.8	31.4 ± 3.9
Needle	Sternal	ESt	13.1 ± 1.4	40.3 ± 4.1	42.4 ± 5.0
Needle	Sternal	TSt	10.3 ± 2.1	28.3 ± 4.8	29.0 ± 5.7
Needle	Sternal	HSc	11.6 ± 2.1	29.4 ± 4.0	32.5 ± 4.4
Needle	Lateral	ESt	11.6 ± 1.4	32.4 ± 4.5	33.4 ± 4.3
Needle	Lateral	TSt	10.3 ± 1.8	24.9 ± 4.4	24.5 ± 4.7
Needle	Lateral	HSc	11.6 ± 1.7	29.2 ± 3.7	32.4 ± 4.4
**Electrode type**	**Body position**	**Electrode path**	**Phase angle 5 kHz (°)**	**Phase angle 50 kHz (°)**	**Phase angle 250 kHz (°)**
Clip	Sternal	ESt	3.1 ± 0.5	10.4 ± 1.0	14.4 ± 1.1
Clip	Sternal	TSt	3.1 ± 0.7	9.8 ± 0.8	13.1 ± 0.8
Clip	Sternal	HSc	3.1 ± 0.6	8.3 ± 1.0	11.0 ± 1.0
Clip	Lateral	ESt	3.5 ± 1.1	9.9 ± 0.8	12.8 ± 0.9
Clip	Lateral	TSt	3.9 ± 1.1	9.3 ± 1.1	11.0 ± 1.0
Clip	Lateral	HSc	2.9 ± 0.6	8.1 ± 1.2	10.7 ± 1.1
Needle	Sternal	ESt	2.9 ± 0.3	10.5 ± 0.5	14.4 ± 0.3
Needle	Sternal	TSt	3.0 ± 0.5	9.5 ± 0.8	12.2 ± 1.4
Needle	Sternal	HSc	2.8 ± 0.4	8.0 ± 1.1	10.6 ± 1.4
Needle	Lateral	ESt	3.0 ± 0.3	9.5 ± 0.7	12.4 ± 0.6
Needle	Lateral	TSt	3.2 ± 0.4	8.9 ± 0.7	10.7 ± 0.9
Needle	Lateral	HSc	2.8 ± 0.4	8.1 ± 1.1	10.7 ± 1.4

Electrical characteristics derived from Cole–Cole modeling are provided in S2 Table. Across conditions, Xmax and PAmax were consistently observed between 50 and 250 kHz.

Agreement between electrode types is summarized in Table 4, and the corresponding Bland–Altman plots are presented in S1–S3 Figs. A total of 39 paired observations were included for each frequency after exclusion of three measurements that did not meet the predefined Cole–Cole quality criterion.

**Table 4:** Agreement between clip and needle electrode measurements assessed by Bland–Altman analysis.

Resistance	Bias (95% CI)	Lower limits of agreement (95% CI)	Upper limits of agreement (95% CI)	Percentage limits of agreement	r	*P* value
Frequency						
5 kHz	0.98 (-5.56 to 7.52)	-38.58 (-49.9 to -27.30)	40.53 (29.25 to 51.81)	-18.01 to 20.45	0.862	<0.0001
50 kHz	-0.08 (-5.58 to 5.43)	-33.37 (-42.86 to -23.88)	33.22 (23.72 to 42.71)	-17.84 to 19.30	0.862	<0.0001
250 kHz	-1.86 (-6.40 to 2.67)	-29.28 (-37.10 to -21.47)	25.56 (17.74 to 33.37)	-18.62 to 17.84	0.870	<0.0001
**Reactance**	**Bias** **(95% CI)**	**Lower limits of agreement** **(95% CI)**	**Upper limits of agreement** **(95% CI)**	**Percentage limits of agreement**	**r**	***P* value**
**Frequency**						
5 kHz	1.39 (0.28 to 2.49)	-5.29 (-7.19 to -3.39)	8.06 (6.16 to 9.96)	-48.53 to 74.80	0.386	0.0152
50 kHz	0.83 (-0.28 to 1.94)	-5.86 (-7.77 to -3.95)	7.52 (5.61 to 9.43)	-20.26 to 27.25	0.856	<0.0001
250 kHz	0.72 (-0.44 to 1.89)	-6.32 (-8.33 to -4.31)	7.76 (5.76 to 9.77)	-21.18 to 27.81	0.869	<0.0001
**Phase angle**	**Bias** **(95% CI)**	**Lower limits of agreement** **(95% CI)**	**Upper limits of agreement** **(95% CI)**	**Percentage limits of agreement**	**r**	***P* value**
**Frequency**						
5 kHz	0.32 (0.07 to 0.57)	-1.18 (-1.61 to -0.75)	1.82 (1.39 to 2.25)	-45.19 to 68.19	0.379	0.0173
50 kHz	0.23 (0.05 to 0.42)	-0.89 (-1.21 to -0.57)	1.35 (1.03 to 1.67)	-9.78 to 15.09	0.894	<0.0001
250 kHz	0.37 (0.07 to 0.68)	-1.47 (-2.00 to -0.95)	2.22 (1.69 to 2.74)	-12.79 to 20.10	0.843	<0.0001

Reactance and phase angle at 5 kHz showed the weakest agreement, with wide limits of agreement. For resistance at 5, 50, 250 kHz and for reactance and phase angle at 50 and 250 kHz, mean bias was small and correlations were generally strong, although the percentage limits of agreement for reactance at 50 and 250 kHz were wider than those for resistance and extended beyond 27% on the positive side.

Results of linear mixed-effects models are provided in S3 Table. In HSc, between-dog variation accounted for most of the variance (86.4–93.0%) for most variables, and neither electrode type nor body position showed significant effects except for low-frequency reactance and phase angle. In ESt and TSt, between-dog variation also accounted for most of the variance; however, body position affected several MF-BIA parameters, whereas electrode type did not show significant effects in most models.

## Discussion

This study characterized trunk MF-BIA measurements in anesthetized dogs and compared measurements obtained using clip and needle electrodes under controlled conditions. Across various electrode paths and body positions, both electrode types produced feasible measurements with low intra-individual variability. For most frequencies and parameters, clip and needle electrodes showed small mean biases, although the limits of agreement varied among parameters. These findings support the potential use of clip electrodes as a non-invasive alternative under standardized measurement conditions, but the two electrode types should not be considered fully interchangeable.

Several measurements were excluded during Cole–Cole processing, indicating that quality control based on the impedance spectrum can identify datasets that may reflect unstable electrode contact or other artifacts [5]. In the present study, excluded datasets were observed in both sternal and lateral recumbency when clip electrodes were used, consistent with the possibility that contact variability can affect spectral consistency. Careful attention to electrode attachment and verification of spectral plausibility may therefore improve data quality. Electrical property analysis based on the Cole–Cole model showed that the maximum reactance and phase angle consistently appeared between 50 and 250 kHz under all conditions. In contrast, reactance and phase angle at 5 kHz were relatively low and exhibited substantial variability. Although these low frequency measurements are generally considered to have limited physiological relevance, our findings further suggest that they may not reliably reflect the capacitive behavior of cell membranes in dogs. Moreover, although mean biases were small for most impedance parameters, reactance and phase angle at 5 kHz showed weak correlations and wide limits of agreement.

In this study, reactance and phase angle at 250 kHz were higher than those at 50 kHz. Reactance and phase angle are commonly evaluated at 50 kHz, and their values tend to be close to the maximum at this frequency in humans [1,5,20]. An in vitro study of canine skeletal muscle also showed that reactance exhibited near-maximum values at 50 kHz [1]. Consistently, in vivo canine studies on body composition using a single-frequency bioimpedance analysis have typically been conducted at 50 kHz [11–15]. Furthermore, previous studies on bioimpedance analysis in donor dogs have employed measurements of reactance and phase angle only at 50 kHz, following protocols commonly applied in human research [16]. The frequency range at which reactance or phase angle reaches its maximum value, which reflects the electrical properties of cell membranes, has been reported to vary according to species or objects [2,21–23]. The findings of the present study may reflect species-specific characteristics of MF-BIA in dogs, suggesting that when evaluating reactance or phase angle in dogs, it may be preferable to perform measurements at 250 kHz rather than at 50 kHz in the current setting. In humans, trunk phase angle tends to be higher at 250 kHz than at 50 kHz when compared with hemi-body measurements, including the limbs [5,20], suggesting that this may also reflect characteristics related to electrode placement during the present study setting.

While previous studies in dogs have commonly used measurements between the elbow and stifle [13,14,16–18], the present study evaluated three different electrode paths on the trunk: ESt, TSt, and HSc. Measurements were feasible at all electrode paths; however, values obtained at TSt in lateral recumbency tended to show greater inter-individual variability. Moreover, measurements at ESt and TSt may be susceptible to positional effects. In this study, a cotton towel was placed between the limbs to prevent contact and crossing, thereby minimizing any potential influence on the measured values. These differences may be partially explained by variations in segment length, cross-sectional area, and body composition at each site [1,2]. We speculate that electrode paths involving the stifle may lead to higher variability in measurements in lateral recumbency than in sternal recumbency. Therefore, sternal recumbency may be preferable to lateral recumbency for trunk MF-BIA in dogs, particularly when the stifle is included in the electrode path, to minimize measurement variability.

Resistance at 50 kHz, as well as at R0 and at Rinf, especially at the ESt and HSc sites, was consistent with previous reports [16,18], regardless of electrode type or body position. However, other studies measuring resistance in the trunk region have reported lower values, with a mean of approximately 130 Ω [17], possibly due to differences in measurement protocols or dog populations. In contrast, reactance and phase angle at 50 kHz obtained in this study were higher than those reported in previous studies [18]. At the TSt site, resistance tended to be lower than at the ESt and HSc sites, suggesting that the electrode path can affect the MF-BIA results. Factors such as breed, age, segment geometry, and body composition may contribute to inter-individual variability, making it difficult to define absolute reference values [12]. The present study demonstrated relatively high inter-individual variability, with most of the variation in measurements attributable to individual-specific factors.

Despite these sources of variability, MF-BIA showed excellent repeatability, with intra-individual coefficients of variation below 1% under controlled conditions. The effect of movement at the measurement site due to mechanical ventilation on the measured values in anesthetized dogs was considered minimal. In the present study, no significant effect of electrode type was detected on the measured values, except for reactance at 50 kHz in the TSt group. Strong correlations were observed between clip and needle electrodes for resistance (5, 50, and 250 kHz), as well as for reactance and phase angle (50 and 250 kHz). Mean biases were small, although the percentage limits of agreement commonly extended to approximately 10–20% and exceeded this range for some reactance measurements. MF-BIA using clip electrodes, which are non-invasive, may provide a useful clinical tool for case-by-case monitoring of body fluid balance and cell membrane stability, especially when evaluating trends over time in individual dogs.

This study has some limitations. The sample size was small and limited to Beagle dogs, and sex-related effects could not be evaluated. Measurements were obtained under general anesthesia with neuromuscular blockade to minimize motion; therefore, outcomes under conscious conditions remain to be evaluated for clinical translation. In addition, mild laboratory abnormalities in some dogs may have contributed to biological heterogeneity. Despite these limitations, the controlled design allowed assessment of repeatability and agreement across electrode types and conditions.

## Conclusions

Trunk MF-BIA measurements obtained using clip electrodes were highly repeatable and showed small mean biases relative to those obtained using needle electrodes in anesthetized dogs, although the limits of agreement varied among parameters and were particularly wide for reactance and phase angle at 5 kHz. MF-BIA using clip electrodes may be useful for non-invasive monitoring of longitudinal trends within individual dogs; however, electrode placement site and body position influenced MF-BIA measurements and should be standardized or carefully accounted for, particularly in lateral recumbency when the stifle is included in the electrode path. Together, these findings support the potential clinical utility of MF-BIA for monitoring body fluid status and membrane integrity in dogs, while highlighting the importance of standardized measurement conditions.

## Supporting information

S1 Table Multifrequency bioimpedance parameters and analysis status for individual dogs. Values represent the mean of 150 repeated measurements obtained at 50-ms intervals for each dog and measurement condition. R0, resistance at zero frequency; Rinf, resistance at infinite frequency; Xmax, maximum reactance; PAmax, maximum phase angle; ESt, from the elbow to the stifle; TSt, from the level of first thoracic spinous process to the stifle; HSc, from the vertex of the head to the level of sacral spinous process. NA, not available. Measurements marked “Excluded” did not meet the predefined Cole–Cole quality criterion because the resistance at the center of the Cole–Cole circle was outside the range defined by the resistance values at 5 and 250 kHz; corresponding Cole–Cole-derived parameters are shown as NA.
(XLSX)

S2 Table Electrical characteristics derived from multifrequency bioimpedance analysis using the Cole–Cole model. Values are presented as mean ± standard deviation. R0, resistance at zero frequency; Rinf, resistance at infinite frequency; Xmax, maximum reactance; PAmax, maximum phase angle; ESt, from the elbow to the stifle; TSt, from the level of first thoracic spinous process to the stifle; HSc, from the vertex of the head to the level of sacral spinous process.
(DOCX)

S3 Table Results of linear mixed-effects model for multifrequency bioimpedance parameters. Linear mixed-effects models were used to evaluate the effects of electrode type and body position. Effect of individual dogs (ID) was included as a random effect. ESt, from the elbow to the stifle; TSt, from the level of first thoracic spinous process to the stifle; HSc, from the vertex of the head to the level of sacral spinous process; Effect of ID, individual contribution to the total variance.
(XLSX)

S1 Fig Bland–Altman plots comparing resistance measurements obtained using clip and needle electrodes. Panels A, B, and C show measurements obtained at 5, 50, and 250 kHz, respectively. Differences were calculated as clip minus needle measurements. The central solid line indicates the mean difference (bias), the dashed lines indicate the 95% limits of agreement, and the shaded areas indicate the corresponding 95% confidence intervals.
(PNG)

S2 Fig Bland–Altman plots comparing reactance measurements obtained using clip and needle electrodes. Panels A, B, and C show measurements obtained at 5, 50, and 250 kHz, respectively. Differences were calculated as clip minus needle measurements. The central solid line indicates the mean difference (bias), the dashed lines indicate the 95% limits of agreement, and the shaded areas indicate the corresponding 95% confidence intervals.
(PNG)

S3 Fig Bland–Altman plots comparing phase angle measurements obtained using clip and needle electrodes. Panels A, B, and C show measurements obtained at 5, 50, and 250 kHz, respectively. Differences were calculated as clip minus needle measurements. The central solid line indicates the mean difference (bias), the dashed lines indicate the 95% limits of agreement, and the shaded areas indicate the corresponding 95% confidence intervals.
(PNG)
