# Supplementary material for: Comparison of clip and needle electrodes for multifrequency bioimpedance analysis in anesthetized dogs
Source: PLoS One. 2026 Aug 6;21(8):e0355338. doi: 10.1371/journal.pone.0355338 (PMC13446663; doi:10.1371/journal.pone.0355338)
Supplement: Supplementary file 1 [file pone.0355338.s007.pdf]

The authors present their work, where multifrequency bioimpedance analysis in anesthetized dogs was conducted. The authors highlight the need for this work by noting the lack of data in the reported literature for this area, as well as the need for a noninvasive means to perform these measurements. The authors highlight areas where these measurements can assist in the evaluation of human health and expect this will also have an impact in the evaluation of animal health.

The described protocol is humane and appears to have met the prescribed standard for ethical research. The authors importantly note that the animals used in the study recovered appropriately after completion of the experiments described. The authors explain their approach for performing the measurements, referencing a study performed in humans (PMID: 29330426) and also note several planned methods for statistical analysis.

I recommend that this work be published if the following revisions can be made:

- It would be useful to include this reference in motivating the work performed by the authors: https://doi.org/10.1111/j.1476-4431.2009.00457.x
- The presentation style for the measured data presented in the main paper and supplemental data) made it difficult for me to ascertain the measurements performed on each dog. It would be useful to include the measurements performed for each dog for readers to be able to review, in the supplemental data section.
- Noting the short title for the paper (Clip vs needle electrodes for MF-BIA in dogs), it would be useful to report the details of the Bland-Altman analysis to confirm that measurements using clip vs needle electrodes are indeed similar. Several options for reporting standards for a Bland-Altman analysis are offered in Table 2 of this paper (https://doi.org/10.3390/diagnostics10050334)
