# Supplementary material for: Comparison of clip and needle electrodes for multifrequency bioimpedance analysis in anesthetized dogs
Source: PLoS One. 2026 Aug 6;21(8):e0355338. doi: 10.1371/journal.pone.0355338 (PMC13446663; doi:10.1371/journal.pone.0355338)
Supplement: Supplementary file 2 [file pone.0355338.s002.docx]

S2 Table. Electrical characteristics derived from multifrequency bioimpedance analysis using the Cole–Cole model.

| **Electrode type** | **Body position** | **Electrode site** | **R0 (Ω)** | **Rinf (Ω)** | **Xmax (Ω)** | **PAmax (°)** |
| --- | --- | --- | --- | --- | --- | --- |
| **Clip** | **Sternal** | **ESt** | 263.6 ± 31.1 | 111.4 ± 9.5 | 45.5 ± 7.0 | 13.6 ± 0.9 |
| **Clip** | **Sternal** | **TSt** | 210.4 ± 24.7 | 93.7 ± 8.1 | 33.8 ± 5.3 | 12.5 ± 0.8 |
| **Clip** | **Sternal** | **HSc** | 249.8 ± 23.4 | 114.8 ± 7.3 | 34.1 ± 3.7 | 10.6 ± 0.9 |
| **Clip** | **Lateral** | **ESt** | 237.4 ± 29.9 | 106.7 ± 11.1 | 37.4 ± 5.8 | 12.2 ± 0.8 |
| **Clip** | **Lateral** | **TSt** | 203.5 ± 38.5 | 91.1 ± 9.3 | 27.8 ± 5.4 | 10.7 ± 1.0 |
| **Clip** | **Lateral** | **HSc** | 241.6 ± 27.4 | 117.7 ± 19.5 | 32.6 ± 3.4 | 10.4 ± 0.9 |
| **Needle** | **Sternal** | **ESt** | 263.6 ± 26.1 | 114.6 ± 13.0 | 45.9 ± 5.1 | 13.6 ± 0.2 |
| **Needle** | **Sternal** | **TSt** | 203.5 ± 29.7 | 97.8 ± 16.0 | 31.5 ± 5.5 | 11.8 ± 1.1 |
| **Needle** | **Sternal** | **HSc** | 249.4 ± 33.2 | 124.0 ± 23.5 | 33.7 ± 4.2 | 10.3 ± 1.2 |
| **Needle** | **Lateral** | **ESt** | 232.4 ± 28.6 | 108.8 ± 16.0 | 36.0 ± 5.0 | 11.9 ± 0.5 |
| **Needle** | **Lateral** | **TSt** | 193.2 ± 31.4 | 97.5 ± 18.4 | 26.8 ± 4.8 | 10.5 ± 0.8 |
| **Needle** | **Lateral** | **HSc** | 245.8 ± 25.1 | 119.2 ± 18.4 | 33.5 ± 4.0 | 10.5 ± 1.2 |

Values are presented as mean ± standard deviation.

R0, resistance at zero frequency; Rinf, resistance at infinite frequency; Xmax, maximum reactance; PAmax, maximum phase angle; ESt, from the elbow to the stifle; TSt, from the level of first thoracic spinous process to the stifle; HSc, from the vertex of the head to the level of sacral spinous process.
