# Supplementary material for: Comparison of clip and needle electrodes for multifrequency bioimpedance analysis in anesthetized dogs
Source: PLoS One. 2026 Aug 6;21(8):e0355338. doi: 10.1371/journal.pone.0355338 (PMC13446663; doi:10.1371/journal.pone.0355338)
Supplement: Supplementary file 3 [file pone.0355338.s009.docx]

**Response to Reviewer**

**Manuscript title: Comparison of clip and needle electrodes for multifrequency bioimpedance analysis in anesthetized dogs**

**Manuscript ID: PONE-D-26-05274**

We sincerely thank you for your careful evaluation of our manuscript and for your constructive and helpful comments. We have addressed each comment and revised the manuscript accordingly. Our point-by-point responses are provided below. All changes are indicated in the revised manuscript with track, and a clean version has also been submitted.

*- It would be useful to include this reference in motivating the work performed by the authors: https://doi.org/10.1111/j.1476-4431.2009.00457.x*

→ Thank you for suggesting this relevant reference. We have added it to the Introduction to further support the clinical importance of assessing alterations in body fluid distribution in dogs.

*- The presentation style for the measured data presented in the main paper and supplemental data made it difficult for me to ascertain the measurements performed on each dog. It would be useful to include the measurements performed for each dog for readers to be able to review, in the supplemental data section.*

→ Thank you for this helpful suggestion. We have added a new Supporting Information file (S1 Table) containing the MF-BIA parameters for individual dogs. The dataset includes the values obtained for each dog according to electrode type, body position, electrode path, and measurement frequency, as well as the Cole–Cole-derived parameters and the status of any excluded measurements. We have also revised the Results and Data Availability Statement accordingly.

*- Noting the short title for the paper (Clip vs needle electrodes for MF-BIA in dogs), it would be useful to report the details of the Bland-Altman analysis to confirm that measurements using clip vs needle electrodes are indeed similar. Several options for reporting standards for a Bland-Altman analysis are offered in Table 2 of this paper (https://doi.org/10.3390/diagnostics10050334)*

→ Thank you for this important comment. We have expanded the Bland–Altman analysis and its reporting. For each dog, body position, and electrode path, the mean value obtained using clip electrodes was paired with the corresponding mean value obtained using needle electrodes, and differences were calculated as clip minus needle measurements. We now report the bias, lower and upper 95% limits of agreement, their corresponding 95% confidence intervals, and percentage limits of agreement. A total of 39 paired observations were included for each frequency. The results are presented in the new Table 4, and the corresponding Bland–Altman plots have been added as S1–S3 Figures. We also revised the Abstract, Results, Discussion, and Conclusions to clarify that mean biases were small for most parameters, whereas the limits of agreement varied and were particularly wide for reactance and phase angle at 5 kHz. Therefore, the two electrode types should not be considered fully interchangeable.
